# Long-term follow-up of nonspecific small bowel ulcers with a benign course and no requirement for surgery: is this a distinct group?

**DOI:** 10.1186/1471-230X-11-51

**Published:** 2011-05-10

**Authors:** Weifeng Wang, Zhanbo Wang, Yunsheng Yang, Enqiang Linghu, Zhongsheng Lu

**Affiliations:** 1 Department of Gastroenterology and Hepatology, Chinese PLA General Hospital & Chinese PLA Postgraduate Medical School, Beijing 100853, China; 2 Department of Pathology, Chinese PLA General Hospital, Beijing 100853, China

## Abstract

**Background:**

Nonspecific small bowel ulcers are rare and surgical intervention is often believed to be elective. Since the extensive investigation of the small bowel in the 1990s, there have been limited reports of these ulcers and the updates have been unsatisfactory. The aim of this study was to explore the clinical features and natural histories of nonspecific small bowel ulcers through prospective observational study.

**Methods:**

We reviewed the medical records of all patients who had undergone ileocolonoscopy or enteroscopy between 2000 and 2005 in a tertiary referral hospital. Seven patients with small bowel ulcers of unknown cause were identified. These patients were prospectively followed in a prolonged observation until March 2010.

**Results:**

All seven patients (mean age: 54.7 years) presented with mild gastrointestinal symptoms, including chronic diarrhea and/or abdominal pain/discomfort, except for one who was asymptomatic when surveyed for colon polyps. Most patients were suspected of having functional bowel disorders for a long time (4.4 years) before small bowel ulcers were demonstrated on ileoscopy. The ulcers were characteristically multiple, superficial, and small (3-6 mm), locating at the terminal ileum and/or ileocecal valve. Various empirical treatments were applied, and most patients felt partly improved, even relieved. However the gastrointestinal symptoms did not always correlate with the presence of ulcers, and the ulcers tended to be either persistent (4 patients) or recurrent (1 patient). Ileocolonoscopy was repeated 4.1 times during follow-up, even after the lesions had healed. The characteristics of the ulcers, if still present during follow-up, were similar to their earlier characteristics. No patient experienced exacerbation or complications, such as intestinal obstruction, perforation, or bleeding. All patient survived and no surgical intervention was involved during the prolonged follow-up (7.0 years).

**Conclusion:**

The reported patients with nonspecific small bowel ulcers experienced benign courses, inconsistent with previous reports. Without extensive investigation, this disease can be confused with functional bowel disorders.

## Background

Patients with nonspecific small bowel ulcers are rare [1-4]. As indicated by its nomenclature, the diagnosis can only be established after the exclusion of all other possible causes of small bowel ulcer. The previous literature has shown that it is difficult to diagnose nonspecific small bowel ulcers preoperatively because nearly all cases are identified with laparotomy or during the autopsy [3]. The mortality rate for nonspecific small bowel ulcers is reported to be as high as 8.5% [1].

Many case reports of nonspecific small bowel ulcers were presented before 1990 [1,2,5-9], but only a limited number of studies [4,10-12] thereafter. It is noteworthy that in recent decades, the awareness of ileoscopy at colonoscopy has been increasing and new investigative methods have been widely integrated into the investigation of the small intestine, including balloon enteroscopy and capsule enteroscopy [13-15]. This technical progress has facilitated further insight into the various small intestine diseases, including nonspecific small bowel ulcers. However, the updates on nonspecific small bowel ulcers are not yet satisfactory. The features of nonspecific small bowel ulcers are not fully understood, and it is still unclear whether nonspecific ulcers of the small intestine constitute a clinical entity. Here, we present a case series of nonspecific small bowel ulcers treated without surgery during a prolonged follow-up, which manifested clinical courses distinct from those reported previously.

## Methods

### Subjects

We reviewed the medical records of all patients who had undergone either balloon enteroscopy or ileocolonoscopy in 2000-2005 at the Chinese PLA General Hospital, a tertiary referral center located in Beijing, China. The indications for investigations in all patients who underwent ileocolonoscopy and enteroscopy are as follows: abdominal discomfort or pain, constipation, diarrhea, gastrointestinal bleeding, adenoma follow-up, screening for colorectal cancer, health check-up and miscellaneous indications. The medical records for all patients were available in an electronic form. The disease of seven patients was identified as complying with the definition of nonspecific small bowel ulcers. All the subjects presented with ulceration that was limited to the small intestine (including the ileocecal valve), with no involvement of the colon, and extensive investigations excluded the following causes mentioned in previous studies [4,11]: infections (HIV, tuberculosis, cytomegalovirus, etc), neoplasm, drugs (enteric-coated potassium or non-steroidal anti-inflammatory drugs [NSAIDs] used before the first visit), celiac disease, systemic diseases, vascular diseases, radiation, inflammation (inflammatory bowel disease, eosinophilic enteritis), malabsorption, etc. Consistent with previous reports, ulcers were defined as being no smaller than 3 mm in diameter, with significant depth [16]. We measured the size of the ulcers using open biopsy forceps when taking routine biopsies. The Ethics Committee of the Chinese PLA General Hospital approved the protocol of the study.

### Follow-up

All the patients complied with the long-term follow-up visits. During follow-up, the necessary investigations were repeated, including various laboratory studies, a small-bowel radiological study, ileoscopy or enteroscopy, biopsy, and a subsequent pathological evaluation.

All the follow-up data were also kept in our medical record system, except those of one patient who transferred her health care to another hospital. Extra follow-up telephone calls made in March 2010 to confirm the current health status of all the patients.

### Pathological evaluation

A routine biopsy was usually taken from the edge of ulcers once small bowel ulcer was identified with enteroscopy or ileoscopy. The biopsy specimens were kept in formalin and processed with routine hematoxylin-eosin staining. Microscopic observations were interpreted based on a routine histopathology protocol. The modified Marsh classification [17] was used to differentiate the ulcers from celiac disease. Immunohistochemical staining or acid-fast staining was used when lymphoma, tuberculosis, etc. were suspected.

### Statistics

Descriptive statistics were used. Quantitative variables, including age, duration of symptoms, etc., were expressed as means ± SE. All analyses were performed with SPSS version 15.0 (SPSS, Inc., Chicago, IL).

## Results

### Clinical features

The seven patients with nonspecific small bowel ulcers included two women and five men, with a mean age of 54.7 ± 4.0 years (range, 44-76 years) at the initial diagnosis. The clinical features at the initial diagnosis are summarized in Table 1. The major indications for colonoscopy were diarrhea and/or abdominal pain/discomfort. Most patients complained of mild gastrointestinal symptoms listed above. None of them presented with weight loss, anemia, fever, melena, hematochezia, malabsorption, etc. Most patients (6 out 7 cases) suffered from their symptoms for 4.4 ± 2.0 years (range, 0.5-11 years) and considered as functional bowel disorders before small bowel ulcers were demonstrated. Only in one patient (case 7) were ileal ulcers accidentally found during a surveillance colonoscopy for polyps. Extensive investigations were performed in all patients after ulcers were found and all the results were negative. Various empirical treatments were applied, including probiotics and 5-asalazine (5-ASA), except in two patients for whom no medication was prescribed. One patient (case 5) felt relieved after the treatment. Meanwhile three reported that their symptoms were partly improved by probiotics or Claritin, but not 5-asalazine. It is noteworthy that the gastrointestinal symptoms did not always correlate with the presence of ulcers. When the ulcers persisted, the symptoms often remained unchanged or only partially improved, except in one patient (case 7), who remained symptom free. When the ulcers had healed, the symptoms did not necessarily resolve automatically, as in case 2. During follow-up, two patients started to take aspirin for several years. One patient (case 6) started aspirin after the recurrent small bowel ulcers had healed with no more recurrence. Another patient (case 4) commenced aspirin when his nonspecific small bowel ulcers persisted and the ulcers remained unchanged on subsequent endoscopy.

**Table 1:** Clinical feature of patients with nonspecific small-bowel ulcers

	Sex	Age (years)	Duration of symptom(s) before diagnosis (years)	Indication for colonoscopy	Treatment	Duration of follow-up (years)	Outcome (symptom)	Outcome (endoscopy)
Case 1	Male	47	3	Diarrhea plus abdominal pain	Probiotics	8	Partly improved	Persistent
Case 2	Male	61	11	Abdominal pain	None	9	Partly improved	Self-limiting
Case 3	Female	50	1	Abdominal pain plus bloating	Probiotics	6	Persistent	Persistent
Case 4	Male	53	10	Diarrhea plus abdominal discomfort	5-ASA, probiotics and Claritin	6	Partly improved	Persistent
Case 5	Male	52	1	Abdominal pain	Herbal medicine and probiotics	8	Asymptomatic	Self-limiting
Case 6	Female	44	0.5	Diarrhea plus abdominal discomfort	5-ASA and probiotics	5	Asymptomatic	Recurrent
Case 7	Male	76	*	Surveillance for polyps	None	7	*	Persistent

5-ASA: 5-asalazine

* This patient was asymptomatic and ileal ulcers were accidentally found during a surveillance colonoscopy for polyps.

### Endoscopic and histopathological findings

All the ulcers were identified by ileoscopy on the first visit, when terminal ileum intubations (10-25 cm) were performed routinely during colonoscopy. Ileoscopy was repeated 4.1 ± 0.5 times during follow-up, even after the lesions had healed.

#### Macroscopic characteristics of the ulcers

**Figure 1:**
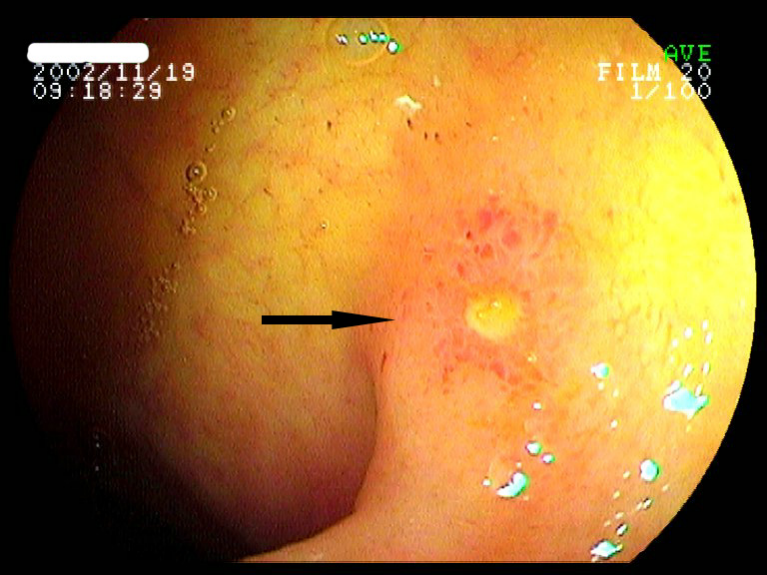
**Ileoscopy revealed superficial circular ulcers in the terminal ileum**.

The diagnostic findings of the first endoscopic procedure are listed in Table 2. All the ulcers were located in the terminal ileum, within 10 cm distal to the ileocecal valve, with or without ileocecal valve involvement (Figure 1). The lesions were characteristically multiple, superficial, and small (greatest dimension 3.7 ± 0.5 mm; range, 3-6 mm), with demarcated margins and clean surfaces. The ulcers were predominantly circular in shape. No deformity or stenosis was found in the terminal ileum or the ileocecal valve. In one patient (case 3), single ulcer was found in the ileocecal valve, with no involvement of the terminal ileum.

**Table 2:** Diagnostic findings of the first endoscopy

	Location of the ulcers	Single or multiple ulcers	Greatest dimension of ulcers (mm)	Predominant shape of ulcers
Case 1	At the terminal ileum and ileocecal valve	Multiple	6	Circular
Case 2	At the terminal ileum and ileocecal valve	Multiple	3	Irregular
Case 3	At the ileocecal valve	Single	3	Irregular
Case 4	At the terminal ileum and ileocecal valve	Multiple	3	Irregular
Case 5	At the terminal ileum and ileocecal valve	Multiple	3	Circular
Case 6	At the terminal ileum	Multiple	3	Circular
Case 7	At the terminal ileum	Multiple	5	Circular or ovoid

#### Histopathological findings

**Figure 2:**
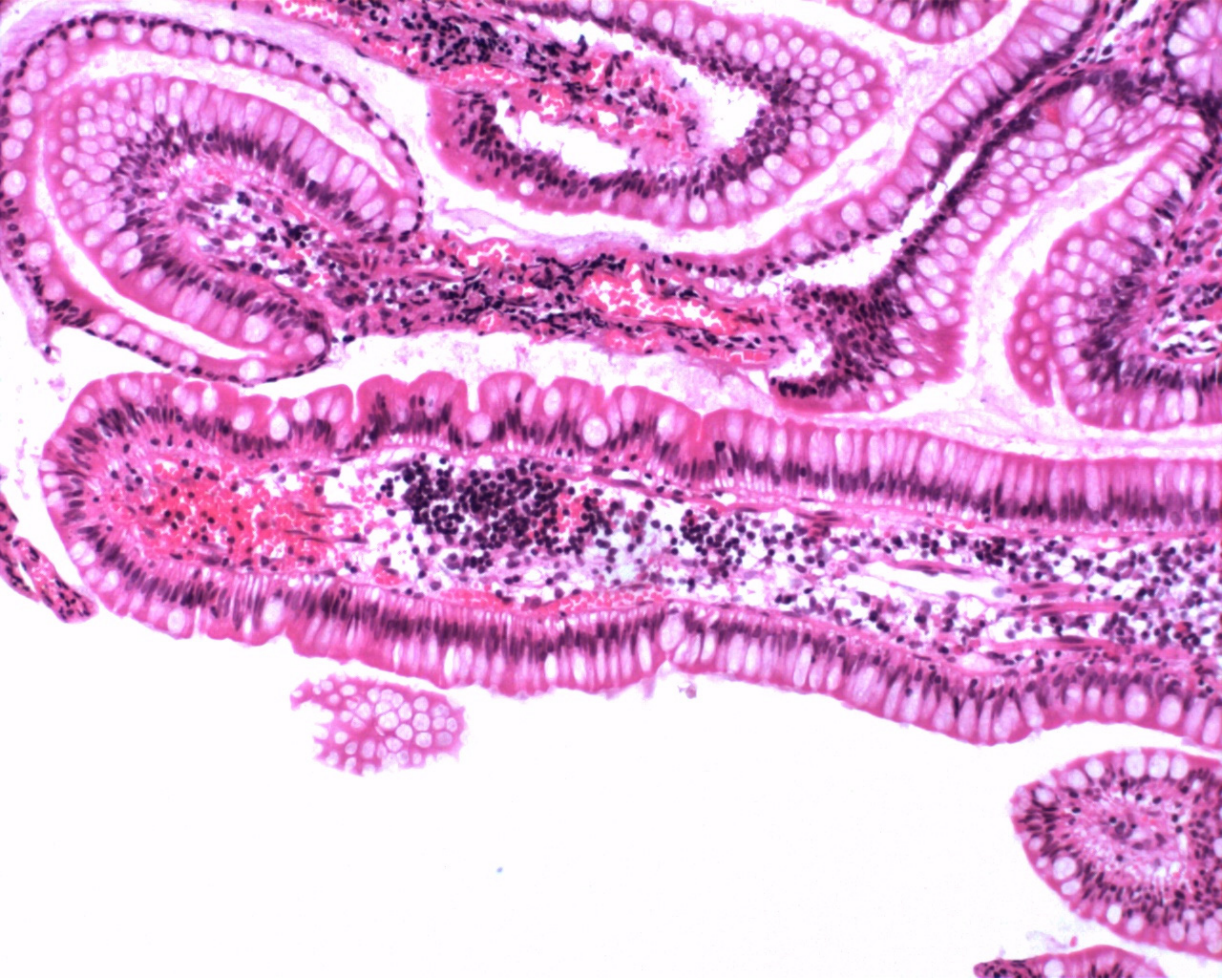
**Microscopic examination of a biopsy specimen showed nonspecific inflammatory infiltration**. Magnification, ×100.

Most patients underwent more than two biopsies and histopathological evaluations (2.4 ± 0.4 times). The histopathological examinations revealed nonspecific inflammation without villous atrophy. Microscopically, there were no granulomas, caseating tissue, eosinophilic infiltration, viral inclusions, etc. (Figure 2). Repeated biopsies showed changes similar to those described above.

#### Repeated endoscopy

During the observation period, all the patients underwent several repeated ileocolonoscopies at specific intervals, as considered suitable by the gastroenterologists. The details of the endoscopic follow-up are shown in Table 3. The characteristics of the ulcers, if still present during follow-up, were similar to their earlier characteristics. On healing, no scars or erythema were found at the site of a previous ulcer. Most patients (5/7) also underwent several gastroscopic examinations, which revealed some minor changes, such as erosion (2/7) or submucosal leiomyoma (1/7), that could not be attributed to the pathogenesis of nonspecific small bowel ulcers. The gastroscopic findings were otherwise normal.

**Table 3:** Endoscopic follow-ups of patients with nonspecific small-bowel ulcers

	No. ileoscopies	Second endoscopy	Third endoscopy	Last endoscopy	No. biopsies	Gastroscopy	Concomitant finding in the colon
Case 1	4	Persistent	Persistent	Persistent	4	Normal	Small single polyp
Case 2	5	Persistent	Persistent	Healing	3	Submucosal leiomyoma of the esophagus was resected endoscopically after the ileal ulcer had healed	Small single polyp
Case 3	2	Persistent	*	*	2	Erosion in the stomach	N/A
Case 4	4	Persistent	Persistent	Persistent	3	Normal	N/A
Case 5	4	Healing	Continued to be normal	Continued to be normal	1	Normal	N/A
Case 6	6	Healing	Recurrent^§^	Healing	2	Normal	N/A
Case 7	4	#	Persistent	#	2	Erosion in the stomach	Small multiple polyps

#Ileoscopy was not attempted during colonoscopy.

* This patient refused a further follow-up colonoscopy.

§Double balloon enteroscopy revealed the same result as the colonoscopy did.

### Outcomes

At the final visit, the outcome was evaluated from both the symptoms and the endoscopic findings. However the endoscopic findings didn't always change in parallel with the symptoms, as shown in Table 1. A final endoscopy showed persistent ulcers in 57.1% of the patients, whereas 14.3% had recurrent ulcers (case 6) and 28.6% had self-limiting ulcers (case 2 and case 5). In case 2 the lesions were shown endoscopically to have disappeared six years after the first endoscopy and eight months later in case 5. In case 6, the ulcers vanished five months after the first endoscopy, but her symptoms, including diarrhea and abdominal discomfort, persisted and a repeated endoscopy 18 months later revealed recurrent ulcers, similar to the previous ulcers. However finally her lesions healed again, and the symptoms disappeared, which can't be attributed to any medication. To date, all the patients have survived for 5-9 years after the prolonged follow-up (7.0 ± 0.5 years). No underlying causes became evident during the observation period. Neither new symptoms nor complications such as strictures of the intestine, ileus, intestinal perforation, or bleeding, appeared. No patient underwent surgical resection of the ulcers during the clinical course.

## Discussion

This series of patients with nonspecific small bowel ulcers manifested specific endoscopic features and benign clinical courses, which is different from previous case reports [2,3,10,18]. Our series was characterized by multiple small ulcers located at the end of the ileum or the ileocecal valve, with mild gastrointestinal symptoms but no bleeding, perforation, or stenosis. The lesions were either self-limiting or persistent. No complications or deterioration were seen in the patients with persistent ulcers, although no special treatment was applied, including surgery.

The average age at the initial diagnosis was 54.7 years, consistent with previous reports [3]. Nonspecific small bowel ulcers affect both sexes, and different reports have shown different representation in the two sexes [2,18]. In our study, there was a clear predominance of males. Most studies have reported long symptomatic histories before the initial diagnosis. Similarly, in the present study, most patients had suffered from gastrointestinal symptoms for 0.5-11 years. Concomitant lesions, including esophageal leiomyoma, erosions of the stomach, and colon polyps, were present but there was no evidence that they correlated with the nonspecific small bowel ulcers.

Indications for further investigation in this study were diarrhea and/or abdominal pain/discomfort, which are different from the indications reported in the literature. Complications resulting from nonspecific small bowel ulcers have often been reported previously, including bleeding, anemia, intestinal obstruction, and perforation [1,2,4,19]. Interestingly, one patient was accidentally diagnosed by ileoscopy during surveillance for colon polyps, and no gastrointestinal symptoms appeared later. Our case series showed good prognoses. Nonspecific small bowel ulcers were persistent in four of the patients presented here, self-limiting in two, and recurrent in only one, but these recurrent ulcers healed again without intervention. Surgical resection is the most common strategy used to treat nonspecific small bowel ulcers, according to the literature [3,4,18]. However, none of our patients showed complications such as perforation, obstruction, bleeding, anemia, etc., so no surgical intervention was required during the 5-9 year observation period. Because no patient underwent surgery, we cannot assess the recurrence rate after surgery in our study. In the literature, some authors describe frequent ulcer recurrence, even after surgery [18], whereas there are also reports that surgery was generally curative [2].

In the literature, three distinct syndromes of nonspecific small bowel ulcers have been proposed [3,18]: 1, isolated nonspecific ulcers [1,10], which are usually located in the distal ileum and are identified by laparotomy for intestinal obstruction and bleeding, etc.; 2, idiopathic chronic ulcerative enteritis, which manifests with fever, diarrhea, or mucosal atrophy and mimics celiac disease [7,11,20] (other terms are also used to describe this condition, such as nongranulomatous chronic idiopathic enterocolitis [21], chronic ulcerative nongranulomatous jejunoileitis or idiopathic chronic ulcerative enteritis [6], and chronic nonspecific ulcerative duodenojejunoileitis [20]); 3, cryptogenetic multifocal ulcerous stenosing enteritis [12,18], usually presenting with more than 20 ulcers in the small bowel and multiple ulcerative obstructions, which often recur after surgery. All these conditions are considered to be nonspecific and no possible causes have been confirmed, although some possible etiologies have been explored, including vasculitis [12]. When all the clinicopathological features are considered, none of the patients in this study can be referred to any of the syndromes cited above. Our case series is similar to the case reported by Borsch et al. [5], which was assumed to be IBS before a diagnosis of nonspecific small bowel ulcers was made. As stated above, the symptoms of most of the patients in this study were compatible with the definition of functional bowel disorders and there were no "alarm" symptoms indicating a need for extensive exploration according to the Rome III criteria for functional gastrointestinal disorders [22]. Most of our patients were assumed to have functional bowel disorders, such as IBS, before ileoscopy. As we know, terminal ileum intubations and subsequent ileoscopy are not routinely performed in colonoscopy practice when no inflammatory bowel disease or ileal lesions are suggested before the colonoscopy, Recent data have shown that the terminal ileum intubation rate is low (17%-21%) during colonoscopy in various practice settings [23]. Actually, ileoscopy is technically feasible and adds only a couple of minutes to the duration of the procedure [24]. Some authors have suggested that routine terminal ileum intubation should be applied for patients with abdominal pain, diarrhea, or anemia to improve the diagnostic yield [25-27]. This study seems to further justify the use of ileoscopy during colonoscopy for patients suspected of functional bowel disorders, including IBS.

It has often been stated in the literature that nonspecific small bowel ulcers are difficult to diagnose preoperatively [1-3]. This preconception is changing, as shown in our report and those of others [5,11], and can be attributed to doctors' increasing awareness of small bowel disease and the substantial progress in visualizing the small intestine by capsule endoscopy and double or single balloon enteroscopy [13,28,29], together with the broad application of ileoscopy during colonoscopy [25,30]. Hence, nonspecific small bowel ulcers can be identified without recourse to surgery.

The causes underlying nonspecific small bowel ulcers remain obscure. Because ileal ulcer is found in various diseases, including infections, neoplasm, inflammatory bowel disease, etc., it is important to explore these possibilities before a diagnosis of nonspecific small bowel ulcers is made. In China, Crohn's disease and intestinal tuberculosis are the most common diseases involving the ileum, and both show granuloma on microscopic evaluation. However, it is always difficult to establish a definite diagnosis of Crohn's disease [31,32]. Therefore, in clinical practice, much attention has been paid to the differentiation of Crohn's disease and intestinal tuberculosis from ileal ulcers. All the patients reported here underwent extensive investigations to rule out Crohn's disease, intestinal tuberculosis, and other possible causes. No underlying disease appeared during the long-term follow-up, which strengthened the initial diagnosis of nonspecific small bowel ulcers. The negative effects of the long-term use of 5-ASA in two patients may reflect the great differences between nonspecific small bowel ulcers and inflammatory bowel disease.

NSAID enteropathy [10,33,34] has been recognized in recent decades as an important cause of small bowel ulcers. Some previously assumed nonspecific small bowel ulcers may have been attributable to chronic NSAID use, especially those with diaphragm changes in the small intestine. In our study, we confirmed that all the patients had not used NSAIDs before their initial diagnosis. Two patients became chronic aspirin users during follow-up. Their final results showed that, in this study, chronic aspirin use did not interfere with the clinical course.

Other than surgery, no effective medication for nonspecific small bowel ulcers has so far been validated [3]. In our study, a variety of empirical medicines were used, including 5-ASA, probiotics, and herbal medicines. Probiotics seemed partly effective in two patients with diarrhea, but they had no direct effect on the ulcer itself. Long-term 5-ASA therapies were prescribed in two patients. However 5-ASA failed in both. Previous data have shown that corticosteroid therapy improves both the symptoms and histology of a certain group of nonspecific small bowel ulcers, called "nongranulomatous chronic idiopathic enterocolitis" [11], whereas another study showed that prednisolone did not benefit these patients [18]. In our study, no patient received corticosteroid therapy for fear that it might increase the risk of perforation. Generally speaking, no medication was found to be effective for nonspecific small bowel ulcers in this study, although some ulcers healed.

## Conclusion

Our results indicate that a distinct group of patients with ileal ulcers show a benign clinical course. Prolonged follow-up showed that these ulcers can be either self-limiting or persistent, but no serious complications appeared, and no surgery was necessary. No efficacious treatment has yet been identified. Nonspecific ulcer of the small intestine is probably a heterogeneous entity, and we have presented here a unique manifestation of it. Because most of our patients were suspected of having functional bowel disorders, including IBS, before the ulcers were identified, it seems worthwhile to perform routine ileoscopy in patients with presumed functional bowel disorders. Further studies from multiple centers are required to confirm this.

## Competing interests

The authors declare that they have no competing interests.

## Authors' contributions

WW conceived the study, collected all clinical data and drafted the manuscript. ZW was responsible for pathological study of biopsy specimens. YY interpreted the data and critically reviewed the manuscript. EL and ZL performed endoscopy procedures and participated in the follow-up. All authors read and approved the final manuscript.

## Pre-publication history

The pre-publication history for this paper can be accessed here:

http://www.biomedcentral.com/1471-230X/11/51/prepub
